# ES cell-derived presomitic mesoderm-like tissues for analysis of synchronized oscillations in the segmentation clock

**DOI:** 10.1242/dev.156836

**Published:** 2018-02-15

**Authors:** Marina Matsumiya, Takehito Tomita, Kumiko Yoshioka-Kobayashi, Akihiro Isomura, Ryoichiro Kageyama

**Affiliations:** 1 Institute for Frontier Life and Medical Sciences, Kyoto University, Kyoto 606-8507, Japan; 2 Graduate School of Biostudies, Kyoto University, Kyoto 606-8501, Japan; 3 Graduate School of Medicine, Kyoto University, Kyoto 606-8501, Japan; 4 Japan Science and Technology Agency, PRESTO, Saitama 332-0012, Japan; 5 Institute for Integrated Cell-Material Sciences, Kyoto University, Kyoto 606-8501, Japan

**Keywords:** Chemical library screening, Embryonic stem cell, Induced presomitic mesoderm, Segmentation clock, Self-organization, Synchronized oscillation

## Abstract

Somites are periodically formed by segmentation of the anterior parts of the presomitic mesoderm (PSM). In the mouse embryo, this periodicity is controlled by the segmentation clock gene *Hes7*, which exhibits wave-like oscillatory expression in the PSM. Despite intensive studies, the exact mechanism of such synchronous oscillatory dynamics of *Hes7* expression still remains to be analyzed. Detailed analysis of the segmentation clock has been hampered because it requires the use of live embryos, and establishment of an *in vitro* culture system would facilitate such analyses. Here, we established a simple and efficient method to generate mouse ES cell-derived PSM-like tissues, in which *Hes7* expression oscillates like traveling waves. In these tissues, *Hes7* oscillation is synchronized between neighboring cells, and the posterior-anterior axis is self-organized as the central-peripheral axis. This method is applicable to chemical-library screening and will facilitate the analysis of the molecular nature of the segmentation clock.

## Abstract (precis)

**Highlighted Article:** Embryonic stem cell-derived presomitic mesoderm-like tissues exhibit synchronized *Hes7* oscillations, anterior-posterior-like axis formation and segment formation, and are suitable for chemical-library screening for detailed analysis of the segmentation clock.

## INTRODUCTION

Somites are periodically formed by segmentation of the anterior parts of the presomitic mesoderm (PSM) under the control of the segmentation clock, the molecular nature of which has been intensively analyzed (Hubaud and Pourquié, 2014; Oates et al., 2012). In the mouse PSM, the expression of the transcriptional repressor Hes7 oscillates synchronously, propagating from the posterior to the anterior region like traveling waves, and each wave leads to somite segmentation (Bessho et al., 2001; Niwa et al., 2011). Hes7 oscillation is regulated by delayed negative feedback: Hes7 protein represses its own expression at delayed timing, and this repression downregulates Hes7 protein expression, leading to reactivation of expression (Bessho et al., 2003; Takashima et al., 2011). Both loss of expression and steady expression of Hes7 lead to severe somite fusion (Bessho et al., 2001; Hirata et al., 2004; Takashima et al., 2011; Sparrow et al., 2012), whereas faster Hes7 oscillation accelerates the pace of segmentation (Harima et al., 2013), indicating that Hes7 oscillation underlies the mouse segmentation clock.

Despite such intensive studies, the exact mechanism of oscillatory dynamics still remains to be analyzed. For example, Hes7 oscillation is synchronized between neighboring PSM cells, but the exact mechanism of the synchronization is not known. It has been shown that Notch signaling is required for synchronized oscillations in the zebrafish PSM: both genetic and pharmacological inhibition of Notch signaling desynchronizes oscillatory expression between neighboring cells, resulting in salt-and-pepper expression patterns (Jiang et al., 2000; Mara et al., 2007; Riedel-Kruse et al., 2007; Özbudak and Lewis, 2008; Delaune et al., 2012). The zebrafish Notch ligand DeltaC is expressed in an oscillatory manner under the control of Her genes, homologues of mouse *Hes7*, and this oscillation has been postulated to drive synchronization by periodic activation of Notch signaling (Horikawa et al., 2006; Giudicelli et al., 2007; Mara et al., 2007; Soza-Ried et al., 2014). In the mouse PSM, the expression of the Notch ligand Delta-like1 (Dll1) also oscillates, suggesting that Notch signaling is involved in synchronized oscillations (Maruhashi et al., 2005; Bone et al., 2014; Shimojo et al., 2016). Indeed, dissociation of mouse PSM cells desynchronizes oscillatory expression (Masamizu et al., 2006), whereas re-aggregation of dissociated mouse PSM cells self-organizes synchronized oscillations in a Notch signaling-dependent manner (Tsiairis and Aulehla, 2016). Furthermore, optogenetic induction of pulsatile Dll1 expression entrains oscillatory expression in neighboring cells (Isomura et al., 2017). However, mathematical modeling suggests that the oscillatory expression could be either in-phase, anti-phase or quenched, depending on the delays in Dll1-Notch signaling transmission between cells (Lewis, 2003; Herrgen et al., 2010; Shimojo et al., 2016), and how in-phase oscillation is established in the PSM remains to be analyzed.

Another feature of oscillatory gene expression is propagation of wave patterns from the posterior-to-anterior PSM, suggesting that the phase of oscillation is shifted along the anterior-posterior axis. It has been shown that the Wnt and Fgf signaling pathways exhibit posterior-to-anterior gradients (Aulehla et al., 2003, 2008; Dubrulle and Pourquié, 2004; Ay et al., 2014), suggesting that these signaling gradients could be involved in the phase shift of oscillations. However, the exact mechanism of how the phase shift is controlled along the anterior-posterior axis also remains to be analyzed.

Detailed analysis of the segmentation clock has been hampered because it requires the use of live embryos. Analysis involves both genetic and pharmacological approaches. In genetic approaches, mutant animals are generated in which gene functions are activated or inactivated, whereas with pharmacological approaches, either embryos or PSM tissues prepared from embryos are used for treatment with chemicals that modulate the activities of genes. Establishment of an *in vitro* culture system would facilitate such analyses, and many attempts have been made to induce PSM-like tissues from embryonic stem (ES) cells (van den Brink et al., 2014; Gouti et al., 2014; Chal et al., 2015; Sudheer et al., 2016). Although PSM-like tissues have been successfully induced from ES cells, there are no reports of wave-like propagation of oscillatory gene expression.

Here, we have established a simple and efficient method to induce PSM-like tissues from mouse ES cells, in which *Hes7* expression oscillates like propagating waves. In these induced PSM-like (iPSM) tissues, *Hes7* oscillation is synchronized between neighboring cells, and the posterior-anterior axis is self-organized as the central-peripheral axis. This method is amenable to chemical and siRNA library screening and will facilitate analyses to enhance our understanding of the molecular nature of the segmentation clock.

## RESULTS

### Formation of *Hes7*-oscillating iPSM tissues from mouse ES cells

**Fig. 1.**
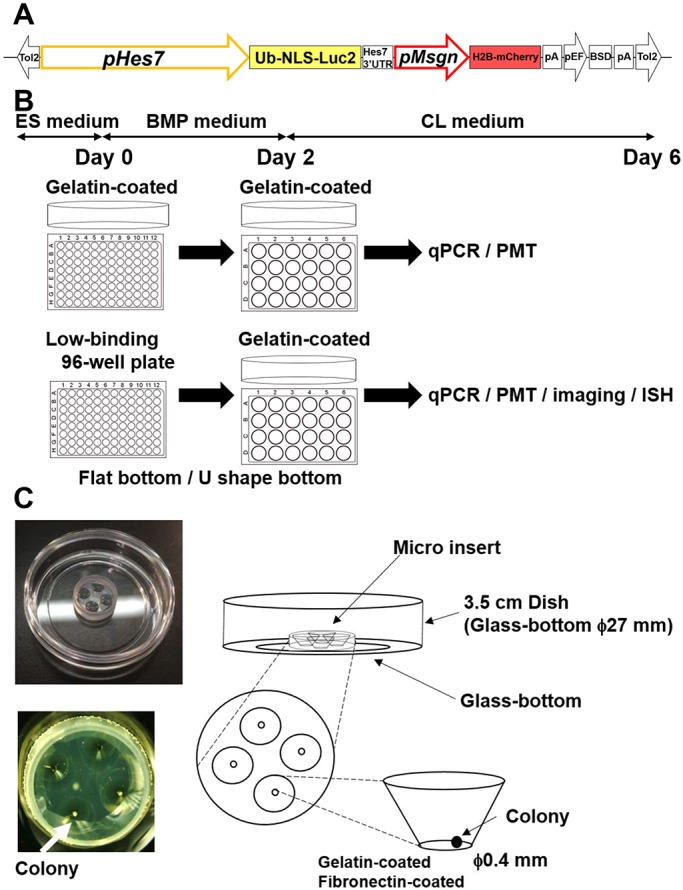
**Strategy for iPSM formation and quantification and imaging of *Hes7* oscillations.** (A) Schematic structure of the *Hes7* and *Msgn1* reporter construct (see Materials and Methods). (B) Culture methods for PSM-like tissue formation from ES cells. (C) Culture method for imaging *Hes7* expression in iPSM colonies. See also Fig. S1.

To monitor *Hes7* expression dynamics, a destabilized luciferase reporter under the control of the *Hes7* promoter (pHes7-Ub-NLS-Luc2), which successfully reported oscillatory expression in the PSM (Takashima et al., 2011), was introduced into ES cells (Fig. 1A). Furthermore, to monitor the efficiency of PSM-like tissue formation, the mesogenin 1 (*Msgn1*) reporter (pMesogenin1-histoneH2B-mCherry) was introduced into ES cells (Fig. 1A). To induce PSM-like tissues from mouse ES cells, we first used the method reported by Chal et al. (2015). These ES cells were cultured in the presence of BMP (BMP medium, see Materials and Methods) for 2 days, and then changed to medium containing the GSK3β inhibitor CHIRON99021 and the BMP antagonist LDN193189 (CL medium, see Materials and Methods) (Fig. 1B). When the cells were cultured in gelatin-coated feeder-free 96-well plates, they were expanded in a monolayer, as described previously (Chal et al., 2015). Under this condition, the expression of *Hes7* and *Msgn1*, as well as other PSM-specific genes such as brachyury (*T*) and *Tbx6*, were induced at days 3-4 (84-96 h) and downregulated at day 6 (∼144 h), while the ES cell-specific marker *Nanog* was downregulated from day 2 (48-60 h) onwards (Fig. S1). At days 4-6, the somitic genes *Pax3* and *Uncx4.1* were also expressed (Fig. S1). To see whether *Hes7* expression oscillates, we monitored *Hes7*-promoter-driven luciferase activity using a highly sensitive photo-multiplier tube (PMT). However, we did not observe clear oscillatory expression of *Hes7* in these cells from day 4 (Fig. S2A). We noticed that *Hes7*-expressing cells were scattered but not clustered under this condition, suggesting that scattered *Hes7* expression may hamper synchronized oscillation.

Because floating culture of embryoid body-like aggregates is often used for organoid formation (Eiraku et al., 2011), we next cultured cells in low-cell-adhesion plates for the initial 2 days (∼48 h) to form floating aggregates (Fig. 1B). When 3000 cells per well were plated in 96-well low-cell-adhesion plates with flat bottoms, multiple colonies of various sizes were formed in each well after 2 days. These colonies were then transferred to CL medium in gelatin-coated culture plates. This condition led to the expression of *Hes7* and *Msgn1* as well as other PSM-specific genes at day 4, at comparable levels to using gelatin-coated plates throughout the culture (Fig. S1). Somitic gene expression was also induced at days 5-6 (Fig. S1). To determine whether *Hes7* expression oscillates, we monitored *Hes7*-promoter-driven luciferase activity of these colonies using a PMT from day 4. *Hes7* reporter activity clearly exhibited oscillatory patterns, suggesting that *Hes7* expression oscillates synchronously in these colonies (Fig. S2B). Therefore, we decided to use the floating culture method to generate induced PSM-like tissues (iPSM) from ES cells. The addition of 1% or 2% Matrigel, a gelatinous protein mixture, during floating culture did not make any significant difference to *Hes7* expression (data not shown). We also changed the duration of the floating culture, but shorter (42 h) and longer (72 h) cultures reduced the *Hes7* expression levels (data not shown).

### The effect of the size of iPSM colonies on *Hes7* oscillations

**Fig. 2.**
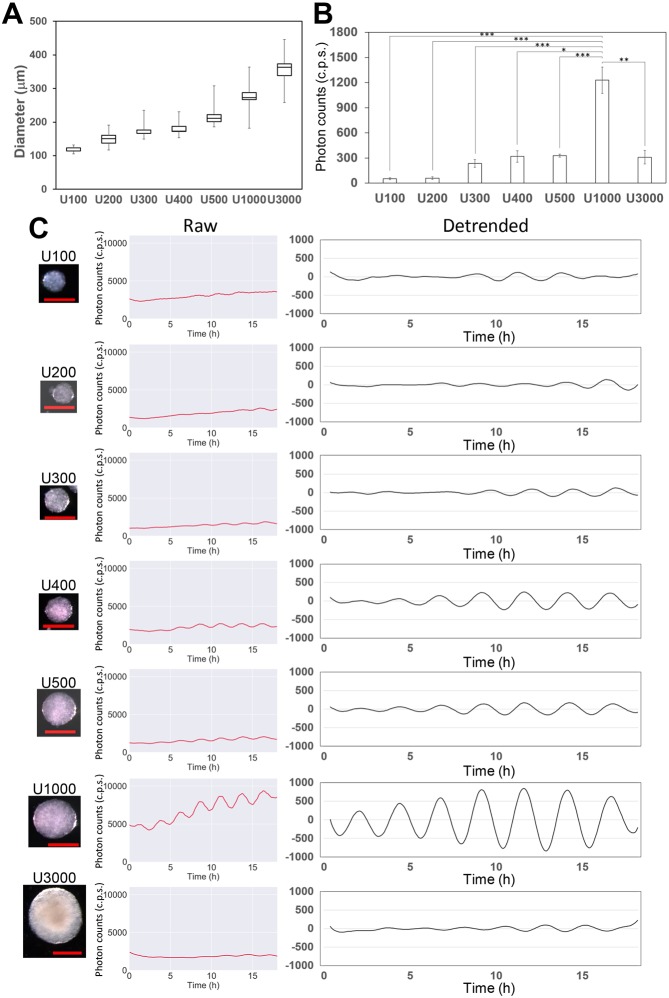
**The effect of the size of iPSM colonies on *Hes7* oscillations.** (A,B) The average diameter and *Hes7* expression of iPSM colonies. One hundred (*n*=16), 200 (*n*=3), 300 (*n*=15), 400 (*n*=9), 500 (*n*=16), 1000 (*n*=22) or 3000 (*n*=3) ES cells seeded per well in low-cell-adhesion 96-well plates with U bottoms were cultured in BMP medium for 2 days. Cells were then transferred to gelatin-coated dishes and cultured in CL medium for 2 days. (A) The diameter of the colonies was measured at day 4. (B) The average amplitudes of *Hes7* oscillations in iPSM colonies. The average amplitudes of three highest pulses of detrended signals from each time series were measured. *Hes7* oscillations in iPSM colonies were monitored by PMT at day 4. *P<0.05, **P<0.01, ***P<0.001, Student's *t*-test. (C) *Hes7* expression in single colonies of varying sizes. The *Hes7* promoter-driven luciferase activities were measured by PMT at day 4. Raw and detrended signals of *Hes7* expression are shown. Only representative data are presented. Representative iPSM colonies are shown on the left. Scale bars: 200 µm. See also Figs S2 and S3.

We next examined the effect of the size of iPSM colonies on *Hes7* oscillations. From day 4 onwards, single colonies of various sizes were separately cultured in 24-well gelatin-coated plates (Fig. S3A), and *Hes7* reporter activity was monitored using a PMT from day 4. We found that all colonies with sizes between ∼100-300 µm exhibited oscillatory expression, and that detrended signals clearly showed robust oscillatory patterns (Fig. S3C). Compared with smaller and larger colonies, medium-size colonies (150-260 µm) tended to exhibit higher amplitudes and more stable oscillations (Fig. S3B,C). To obtain colonies with more uniform sizes, we next used low-cell-adhesion 96-well plates with U bottoms during the first 2 days. Each well formed a single colony of varying sizes, depending on the number of cells seeded (Fig. 2A). When 100-500 or 3000 cells per well were seeded on day 0 (U100-U500 or U3000), we observed *Hes7* oscillations at day 4, but their amplitudes were relatively small (Fig. 2B,C). By contrast, when ∼1000 cells per well were seeded on day 0 (U1000), colonies with the average size of 273.0±3.9 µm were formed at day 4 (Fig. 2A) and exhibited robust *Hes7* oscillations with the highest amplitudes from day 4 onwards (Fig. 2B,C). In these colonies, more than 10 pulses of *Hes7* oscillations with the average period of 167.2±2.2 min occurred, indicating that this condition (U1000) most efficiently produced iPSM tissues. Therefore, we decided to use the U1000 condition for live imaging and quantification of *Hes7* expression.

To perform time-lapse imaging of *Hes7* expression in iPSM tissues, colonies were cultured in gelatin-coated wells of micro inserts (Fig. 1C) or in fibronectin-coated dishes from day 4 onwards. In the former condition, colonies loosely attached to the bottom (and occasionally moved), whereas in the latter condition, they attached to the bottom and expanded.

### Time-lapse imaging of synchronized *Hes7* oscillation in iPSM tissues

**Fig. 3.**
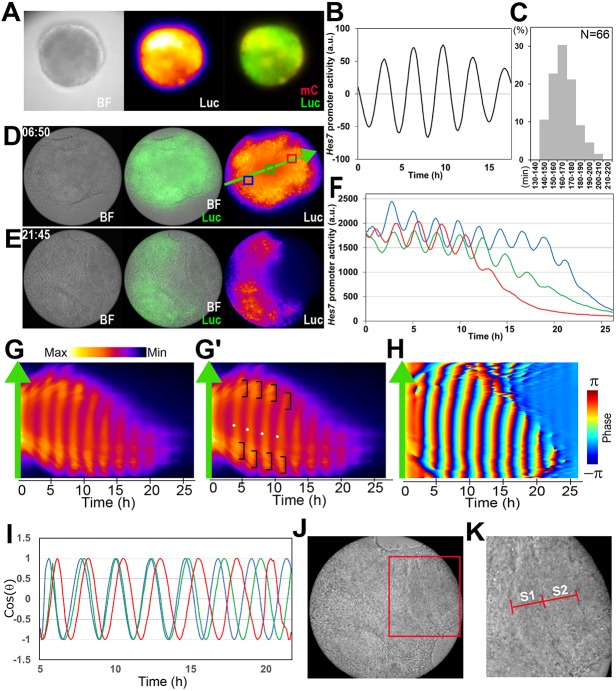
**Live imaging of *Hes7* expression in a single iPSM colony.** iPSM colonies were cultured in either gelatin-coated (A-C, *n*=8) or fibronectin-coated (D-K, *n*=13) dishes. (A) Bright-field (BF) and live imaging of *Hes7* promoter-driven luciferase activity (Luc) in an iPSM colony. mCherry (mC) was expressed under the control of *Msgn1* promoter. (B) Detrended luciferase activity in an iPSM colony. All samples (*n*=8) showed similar oscillatory patterns. (C) The period distribution of *Hes7* oscillations in iPSM colonies. (D,E) Bright-field (BF) and live imaging of luciferase activity (Luc) in an iPSM colony at different time points in culture starting from day 4. (F) Intensity of *Hes7* promoter-driven luciferase activity in an iPSM colony. Blue, green and red lines indicate the intensity measured in blue, green and red boxes, respectively, in D. (G,G′) Kymograph of *Hes7* promoter-driven luciferase activity measured along the green arrow shown in D. *Hes7* waves started from the center of this focus (dots in G′), propagated peripherally, and increased in intensity near the end (brackets in G′). (H) Oscillation phase of *Hes7* promoter-driven luciferase activity in G was calculated. (I) Blue, green and red lines indicate the oscillation phase measured in blue, green and red boxes, respectively, in D. All samples (*n*=13) showed similar oscillatory patterns. (J) Bright-field image of an iPSM colony showing segment formation (boxed). (K) Boxed region in J is enlarged. S1 and S2 indicate a newly formed segment and the one formed before S1, respectively (see also Fig. S4).

For time-lapse imaging of *Hes7* expression in iPSM, single colonies produced by the U1000 procedure were cultured in gelatin-coated wells of micro inserts from day 4 onwards (Figs 1C and 3A). *Hes7* expression was significantly upregulated, forming one apparent focus per colony, and spontaneously began synchronized oscillations in this focus (Fig. 3A,B, Movie 1). Although the period of the oscillations was distributed from 140 to 210 min, the majority (∼74%) were 150-180 min (Fig. 3C). *Hes7* oscillations propagated like waves sweeping from the center towards the periphery of each focus (Movie 1). The size of foci soon reached a maximum, and then gradually shrank, because the peripheral regions of each focus seemed to differentiate into somitic cells. This method was good for quantification of *Hes7* expression in the whole iPSM colony. However, the segmentation process was not clear, and it was somewhat difficult to monitor the propagation patterns because colonies sometimes moved.

To improve the imaging condition for *Hes7* waves, we next cultured iPSM colonies in fibronectin-coated dishes. Under this condition, each colony attached to the bottom and expanded because cells proliferated and spread out. In these expanded colonies, *Hes7* expression was again significantly upregulated, forming one apparent focus per colony, and spontaneously started synchronized oscillations (Fig. 3D-I, Movie 2). In these colonies (*n*=13), *Hes7* oscillations propagated radially like waves from the center towards the periphery of each focus at first. However, the oscillations soon became bi-directional, and one direction usually became dominant (Movie 2). Oscillation phase was delayed in the peripheral region compared with the oscillation center (Fig. 3D,F,I; compared with the blue boxed area, the oscillation phase was delayed in the green boxed area and further delayed in the red boxed area), thereby making wave-propagation patterns (Fig. 3G,H). Analysis of kymograph indicated that in this particular iPSM colony, *Hes7* waves started from the center of focus (white dots in Fig. 3G′), propagated peripherally (both upwards and downwards) and became higher in intensity near the end of oscillation (brackets in Fig. 3G′). This high intensity near the end is reminiscent of *Hes7* upregulation in the S−1 region, a group of cells that form a prospective somite, in the anterior PSM (Niwa et al., 2011). Notably, in the peripheral regions, segmental boarders appeared after each pulse of *Hes7* oscillations (Fig. 3J,K, Movie 2, arrowheads). These results indicated that not only wave-like synchronized *Hes7* oscillations but also segmental boundary formation occurred in iPSM tissues.

To examine the *Hes7* expression at the single-cell level, iPSM colonies were produced from mixtures of ES cells carrying the *Hes7* reporter and wild-type ES cells (1:150). By live-imaging analyses of such iPSM colonies cultured in fibronectin-coated dishes, we were able to monitor the *Hes7* reporter expression at the single-cell resolution (Fig. S4A). However, many of these cells moved actively within colonies, becoming out of focus. Out of 20 single cells that were successfully monitored over 8 h, 18 cells showed oscillatory patterns (Fig. S4B,C), suggesting that most iPSM cells exhibited oscillatory expression.

**Fig. 4.**
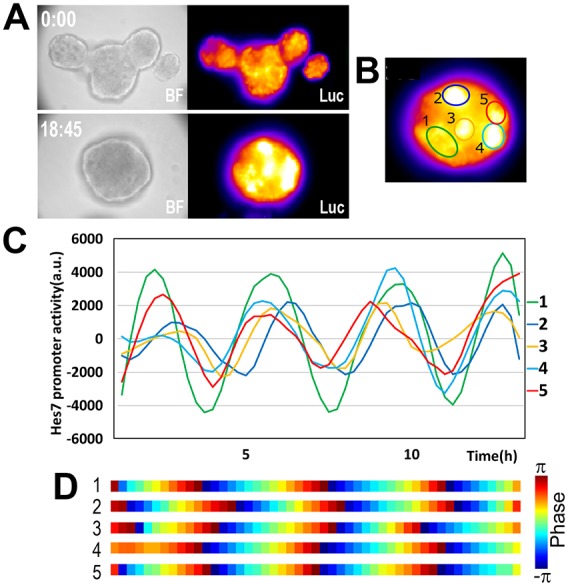
**Live imaging and quantification of *Hes7* expression in fused iPSM colonies.** iPSM colonies were cultured in gelatin-coated dishes. (A) Bright-field (BF) images and luciferase activity (Luc) of fused iPSM colonies at 0:00 and 18:45 in culture starting from day 4. (B) The positions of foci in fused iPSM colonies. (C,D) Quantification of luciferase activities in foci of fused iPSM colonies. Time scale is the same in C and D. *n*=8. All samples showed similar oscillatory patterns.

When multiple colonies were cultured together, they fused to form larger colonies (Fig. 4A). In these fused colonies, multiple foci were present, but some foci fused to form a single focus. Interestingly, *Hes7* oscillations were synchronized in-phase between different foci in such fused colonies (Fig. 4B-D, Movie 3), suggesting that these foci communicate with each other. These oscillatory patterns were very similar to those observed in ePSM generated by reaggregation of dissociated PSM cells from mouse embryos (Tsiairis and Aulehla, 2016).

The synchronized *Hes7* oscillations stopped at day 6. In agreement with this observation, somite markers such as *Pax3* and *Uncx4.1* were expressed at high levels at days 5-6 (Fig. S1). Even when Wnt activity was enhanced by increasing the concentration of CHIRON99021, *Hes7* oscillations did not continue further (data not shown), suggesting that most cells in the foci successively differentiated into somitic tissues after each *Hes7* wave.

### Self-organization of the anterior-posterior axis in iPSM tissues

**Fig. 5.**
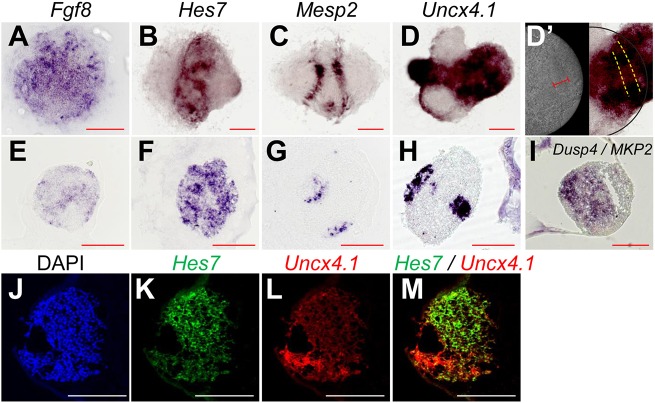
**PSM-specific and somitic gene expression in iPSM colonies.** iPSM colonies produced by the U1000 procedure were cultured on either fibronectin-coated (A-D′) or gelatin-coated (E-M) dishes from day 4. These colonies were subjected to *in situ* hybridization at day 5. (A-I) *In situ* hybridization of *Fgf8* (A,E), *Hes7* (B,F), *Mesp2* (C,G), *Uncx4.1* (D,D′,H) and *Dusp4/MKP2* (I) was performed. D′ shows an enlargement of D (right) and a bright-field image (left). Broken lines indicate segmental borders (D′). (J-M) *In situ* hybridization of *Hes7* (K,M) and *Uncx4.1* (L,M) with DAPI staining (J) was performed. Scale bars: 200 µm.

Wave patterns of *Hes7* oscillations sweeping from the center towards the periphery of each focus suggested that the anterior-posterior axis was self-organized in iPSM colonies. To address this issue, we next examined the expression of genes that are differentially expressed along the anterior-posterior axis. iPSM colonies produced by the U1000 procedure were cultured on either gelatin-coated or fibronectin-coated dishes from day 4, and these colonies were subjected to *in situ* hybridization at day 5. *Fgf8* is highly expressed in the posterior PSM (Dubrulle and Pourquié, 2004), and *Dusp4/MKP2* (MAP kinase phosphatase 2) is also expressed at high levels in the posterior PSM under the control of Fgf signaling (Niwa et al., 2007, 2011). Both *Fgf8* and *Dusp4/MKP2* were expressed at higher levels in the inner regions than in the periphery of foci (Fig. 5A,E,I). Endogenous *Hes7* expression also mainly occurred in the central regions of foci (Fig. 5B,F). By contrast, *Mesp2*, an essential gene for somite segmentation, is normally expressed in a band-like pattern in the prospective somite regions of the anterior PSM (Saga et al., 1997). *Mesp2* expression occurred in a band-like pattern at the periphery of foci (Fig. 5C,G). These results indicate that the central-peripheral axis of iPSM foci corresponds to the posterior-anterior axis of the PSM, suggesting that the posterior-anterior axis is self-organized without the need for artificial gradients of Wnt and Fgf signaling in iPSM colonies. Furthermore, our data suggest that after *Mesp2* expression, the peripheral regions differentiate into somitic cells, agreeing with the above observation that segments were periodically formed.

We also examined the expression of the somitic gene *Uncx4.1*, which is specifically expressed in the posterior half of each somite (Mansouri et al., 1997; Neidhardt et al., 1997). *Uncx4.1* was expressed in the peripheral regions of foci, in which segments were formed (Fig. 5D,D′,H). Indeed, *Uncx4.1*-expressing regions were outside the *Hes7*-expressing domain (Fig. 5J-M). In many iPSM colonies, *Uncx4.1* was expressed in two separate regions (Fig. 5D,H). It was probably because *Hes7* waves mostly propagated in two directions, leading to *Mesp2* expression in two separate regions (Fig. 5C,G). Although *Uncx4.1* expression region was faintly segmented along the morphological segmental boarders, it was not restricted to one half of each segment (Fig. 5D,D′,H). These results suggest that the anterior-posterior patterning within somites does not proceed properly in iPSM tissues.

### Effects of inhibitors on *Hes7* oscillations in iPSM tissues

**Fig. 6.**
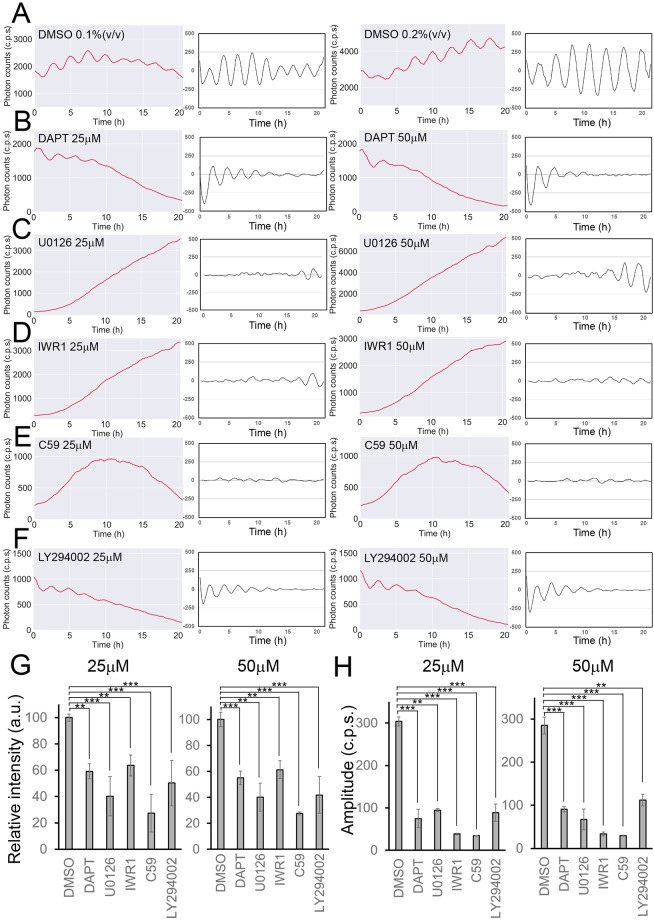
**Effects of inhibitors on *Hes7* oscillations in iPSM colonies.** (A-F) iPSM colonies produced by the U1000 procedure were treated with 0.1% (*n*=6) and 0.2% (*n*=6) DMSO (A), 25 µM (*n*=7) and 50 µM (*n*=7) DAPT (B), 25 µM (*n*=10) and 50 µM (*n*=10) U0126 (C), 25 µM (*n*=7) and 50 µM (*n*=7) IWR1 (D), 25 µM (*n*=7) and 50 µM (*n*=7) C59 (E), and 25 µM (*n*=7) and 50 µM (*n*=7) LY294002 (F) from day 4, and luciferase activity was monitored using a PMT. Both raw and detrended signals are shown. Only representative data are presented. (G,H) Each value represents the average intensity (G) and amplitude (H) with a standard error of *Hes7* reporter expression in iPSM colonies. **P<0.01, ***P<0.001, Student's *t*-test.

Our *in vitro* culture method for iPSM tissue formation is simple and reproducible, and virtually all colonies produced by the U1000 procedure exhibited robust synchronized *Hes7* oscillations. Using iPSM colonies, we next examined the effects of two different concentrations of various inhibitors for the Notch, Fgf and Wnt signaling pathways, all of which are essential for the segmentation clock (Hubaud and Pourquié, 2014; Oates et al., 2012). DAPT (a γ-secretase inhibitor, Dovey et al., 2001) and U0126 (a MEK inhibitor, Favata et al., 1998) inhibit the Notch and Fgf signaling pathways, respectively, and both are required for *Hes7* oscillations in the PSM (Niwa et al., 2011). Both C59 (an inhibitor of porcupine, a membrane-bound O-acyltransferase that is required for Wnt palmitoylation, Proffitt et al., 2013) and IWR1 (a compound that stabilizes Axin2, Chen et al., 2009) inhibit the Wnt pathway. LY294002 (PI3K inhibitor) has been shown to antagonize Wnt signaling activity in the *Xenopus* PSM (Wang et al., 2007). Each of these inhibitors was applied to a single iPSM colony cultured in gelatin-coated dishes at day 4, and *Hes7*-promoter-driven luciferase activity was recorded using a PMT, which can monitor 24-well plates. We found that *Hes7* oscillations in iPSM colonies were quickly dampened in the presence of either DAPT, U0126, IWR1, C59 or LY294002 (Fig. 6B-F), but not in the presence of DMSO only (Fig. 6A), agreeing with previous observations that the oscillatory expression of segmentation clock genes depends on the Notch, Fgf and Wnt signaling pathways (Aulehla et al., 2003; Dale et al., 2003; Niwa et al., 2007; Wahl et al., 2007). Both the intensity of *Hes7* expression and the amplitude of *Hes7* oscillation were reduced by DAPT, U0126, IWR1, C59 or LY294002, compared with DMSO (Fig. 6G,H). These results suggest that the iPSM system is suitable for chemical-library screening to search for new inhibitors that affect synchronized *Hes7* oscillations.

### Chemical-library screening with iPSM tissues

**Fig. 7.**
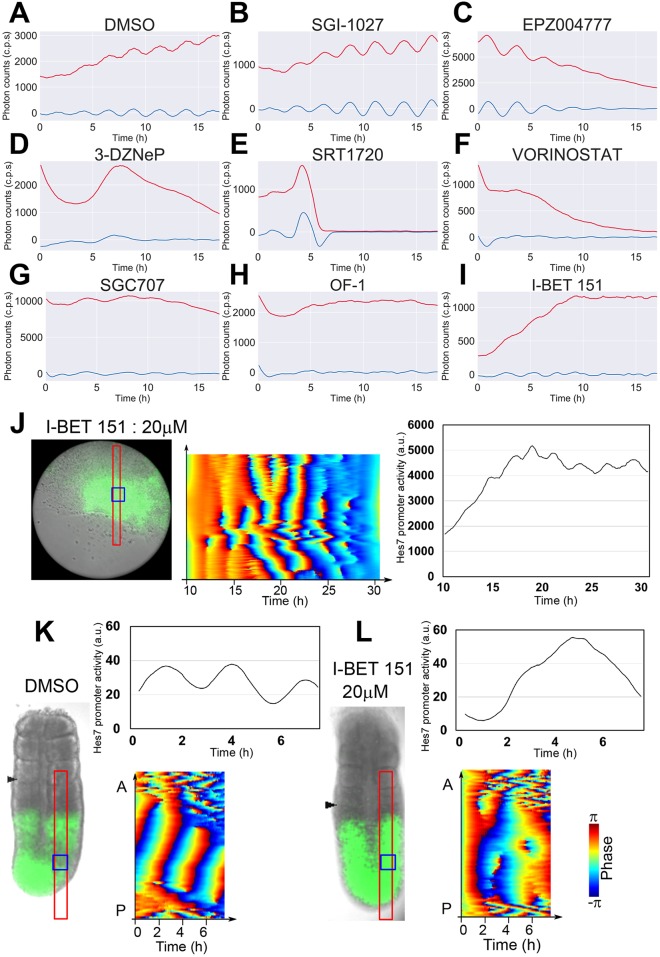
**Chemical-library screening with iPSM colonies and validation with PSM tissues.** (A-I) Chemical-library screening with iPSM colonies. iPSM colonies produced using the U1000 procedure were treated with chemicals from day 4, and *Hes7* promoter-driven luciferase activity was monitored using a PMT. Both raw (red) and detrended (blue) signals are shown. All chemicals (20 µM) were tested in duplicate, which showed similar results. Only representative data are presented. (J) An iPSM colony cultured in a fibronectin-coated dish was treated with 20 µM I-BET 151 from day 4. (Left) Bright-field image and luciferase activity (green). (Middle) Luciferase activity in the red boxed area shown in the left panel was monitored, and the oscillation phase was calculated. (Right) Intensity of luciferase activity in the blue boxed area shown in the left panel was measured. *n*=2. Both samples showed similar oscillatory patterns. (K,L) PSM tissues of E10.5 mouse embryos carrying the *Hes7* reporter were cultured in the presence of either DMSO (K, control, *n*=3) or 20 µM I-BET 151 (L, *n*=3). (Left) Bright-field images and luciferase activity (green). Arrowheads indicate newly formed somite boundaries. (Upper right) Intensity of *Hes7* promoter-driven luciferase activity in the blue boxed regions shown in the left panels was measured. (Lower right) Oscillation phase of *Hes7* promoter-driven luciferase activity in red boxed regions in the left panels was calculated. Phase code for J-L is indicated on the right (see also Fig. S5).

To identify new compounds that affect synchronized *Hes7* oscillations, we next screened a chemical library that contained a set of epigenetic inhibitors (80 compounds). Each inhibitor was added to a single U1000 iPSM colony cultured in gelatin-coated 24-well plates at day 4, and *Hes7*-promoter-driven luciferase activity of each colony was measured using a PMT. We found that some inhibitors, such as the DNA methyltransferase inhibitor SGI-1027, showed no apparent change in *Hes7* oscillations compared with the control (DMSO) (Fig. 7A,B and Fig. S5). However, many inhibitors blocked synchronized *Hes7* oscillations in iPSM colonies, and most of them, such as the histone methyltransferase inhibitors EPZ004777 and 3-deazaneplanocin A (3-DZNeP) and the histone deacetylase (HDAC) inhibitors SRT1720 and VORINOSTAT repressed *Hes7* promoter activity (Fig. 7C-F and Fig. S5, red lines) and thereby blocked *Hes7* oscillations (Fig. 7C-F and Fig. S5, blue lines). Another histone methyltransferase inhibitor, SGC707, also blocked *Hes7* oscillations (Fig. 7G, blue line) but did not repress the *Hes7* promoter activity in iPSM colonies (Fig. 7G, red line). Similarly, OF-1 and I-BET 151, inhibitors of the bromodomain and extra-terminal (BET) family of proteins (Dawson et al., 2011), blocked *Hes7* oscillations (Fig. 7H,I, blue lines) without repressing the *Hes7* promoter activity in iPSM colonies (Fig. 7H,I, red lines). Interestingly, other BET inhibitors, such as PFI-1, CPI203, RVX-000222 and GSK1324726A, also showed similar effects on *Hes7* oscillations (Fig. S5), suggesting that the BET family of proteins are involved in regulation of the segmentation clock.

We further validated the effect of I-BET 151 on *Hes7* oscillations. I-BET 151 was next applied to iPSM colonies cultured in fibronectin-coated dishes. This compound blocked wave propagation patterns without repressing *Hes7* expression (Fig. 7J, Movie 4). In these colonies, although *Hes7* expression was still fluctuating, it became rather steady at the population level (Fig. 7J, right panel). In addition, segment formation did not occur in I-BET 151-treated iPSM colonies. We further examined the effect of I-BET 151 on explant cultures of the PSM prepared from E10.5 mouse embryos carrying the *Hes7* reporter. In the control PSM, *Hes7* expression oscillated in a wave propagation pattern (Fig. 7K, Movie 5). By contrast, *Hes7* expression became steady and non-oscillatory in the I-BET 151-treated PSM (Fig. 7L, Movie 5). Furthermore, somite segmentation was blocked in the presence of I-BET 151 (Movie 5). These results demonstrate that the inhibitor identified by screening with ES cell-derived iPSM colonies produces similar effects on *Hes7* expression in the PSM of mouse embryos, indicating that chemical library screening using the iPSM method is an efficient strategy to find new compounds that affect the segmentation clock.

## DISCUSSION

### The iPSM system is simple and suitable for the analysis of synchronized oscillations

The method for iPSM formation described here is simple, efficient and reproducible, and iPSM colonies are suitable for quantification and imaging of *Hes7* expression. Colonies made by floating cultures exhibited synchronized *Hes7* oscillations with traveling waves. Notably, colonies produced by the U1000 procedure exhibited the highest amplitudes of *Hes7* oscillations. Furthermore, the posterior-anterior axis of the PSM was self-organized as the central-peripheral axis in iPSM colonies, without the need for artificial gradients of Wnt and Fgf signaling. Thus, the key features of *Hes7* oscillations in the PSM autonomously emerge in iPSM colonies. Therefore, this iPSM method facilitates a detailed analysis of *Hes7* expression dynamics to understand how neighboring cells are synchronized (how oscillation phases are coupled), how the central-peripheral axis is self-organized and how wave propagation patterns of oscillations are formed (how oscillation phases are shifted). For the phase-coupling analysis, chimeric mice have been previously produced from eight-cell stage embryos of wild-type and *Lfng*-null mice, which were defective in intercellular coupling of *Hes7* oscillations (Okubo et al., 2012). However, the chimerism ratios of wild-type and mutant cells are difficult to control in such embryos. The iPSM method would allow a similar analysis to be performed more easily and precisely, because mixing ratios of wild-type and mutant ES cells should represent the chimerism ratios. Regarding the posterior-anterior axis formation, it is possible that Fgf and Wnt gradients (high in the posterior and low in the anterior regions) are involved in phase shifting of *Hes7* oscillations, and the involvement of these gradients can be tested by culturing iPSM cells in the presence of higher levels of Fgf and Wnt to determine whether *Hes7* oscillation patterns are modified.

Interestingly, when cultured in fibronectin-coated dishes, colonies expanded and segmental borders appeared at the periphery of iPSM tissues after each pulse of *Hes7* oscillations. Furthermore, *Mesp2* was expressed at the periphery of foci in iPSM colonies. Thus, both synchronized *Hes7* oscillations and segmental boundary formation occurred in iPSM tissues, and therefore this iPSM method is suitable for the analysis of the mechanism of somite segmentation. However, *Uncx4.1* expression was not restricted to a half size of each segment of iPSM tissues, although it occurs specifically in the posterior half of each somite, suggesting that the anterior-posterior patterning within somites does not proceed properly in iPSM tissues.

### The iPSM system is applicable to chemical-library screening

Virtually all iPSM colonies produced by the U1000 procedure exhibited robust *Hes7* oscillations, and therefore this iPSM system can be applied to chemical-library screening to identify new compounds that affect synchronized *Hes7* oscillations. By screening 80 compounds, we found that BET inhibitors such as I-BET 151 and OF-1 inhibited *Hes7* oscillations, leading to rather steady *Hes7* expression in iPSM colonies (Fig. 7J). The effects of I-BET 151 were further validated using explant cultures of PSM prepared from E10.5 mouse embryos carrying the *Hes7* reporter. I-BET 151 was shown to inhibit *Hes7* oscillations in the PSM, leading to steady expression (Fig. 7L). These results indicate that chemical-library screening with iPSM colonies is useful to search for new compounds that affect synchronized *Hes7* oscillations. However, there are still some limits in the iPSM method: although this method showed highly reproducible results, there is some variability in the amplitude, period and patterns of *Hes7* oscillations even in the same iPSM colonies, which may hamper the detection of some effects of chemicals. This variability may depend on the size and shape of iPSM colonies, and how iPSM colonies expand on dishes, and further improvements will be required to reduce such variability.

New compounds found by chemical-library screening will be useful to identify new factors and pathways that regulate *Hes7* oscillations. It has previously been shown that I-BET 151 inhibits transcription through the displacement of the BET family of proteins, such as Brd3 and Brd4, from chromatin (Dawson et al., 2011). Interestingly, other BET inhibitors also reduced the amplitudes of *Hes7* oscillations without repressing *Hes7* expression (Fig. S5). These results suggest that BET proteins could be involved in *Hes7* oscillations. It has previously been reported that *Brd2*-null mice showed neural tube defects (Gyuris et al., 2009; Shang et al., 2009), and that in the absence of *Brd4*, inner cell mass in mouse blastocysts completely degenerated (Houzelstein et al., 2002), indicating essential roles of Brd factors in various steps of embryogenesis. However, the involvement of the BET family of proteins in somite segmentation is not known, and further analysis of these factors will be useful to understand the mechanism of *Hes7* oscillations.

We showed that I-BET 151 led to steady expression of *Hes7* in the PSM at the population level, but it is not clear whether this expression pattern was due to steady *Hes7* expression in all individual cells or loss of synchrony of *Hes7* oscillations between neighboring cells. Imaging of *Hes7* expression at the single cell resolution, as developed for the zebrafish system (Delaune et al., 2012), is required to resolve this issue.

Chemical-library screening with the iPSM system will be also useful to understand the etiology of congenital scoliosis, the most frequent congenital deformity of the vertebrae. It has previously been shown that gene-environment interactions increase the frequency and severity of vertebral defects, and that environmental conditions affect *Hes7* oscillations (Sparrow et al., 2012). Detailed mechanisms of gene-environment interactions remain to be analyzed, and chemical library screening will increase the information about environmental risk factors to the segmentation clock.

### Other applications of the iPSM system

The iPSM method described here will be also useful for other assays. For example, it will be applicable to RNAi screening to identify genes responsible for synchronized *Hes7* oscillations. Furthermore, this system is advantageous for genetic analyses of the segmentation clock, because there is no need to make transgenic animals. CRISPR-Cas9-mediated modification of genes (Koike-Yusa et al., 2014; Shalem et al., 2014; Wang et al., 2014) and introduction of the optogenetic system for spatiotemporal control of gene expression (Isomura et al., 2017) will be easy and fast using this system. Another advantage is that the iPSM method will be suitable for analyzing species-specific differences of the segmentation clock by using either ES or iPS cells. For example, the period of the segmentation clock varies among species, and our method will be useful to analyze the mechanisms for such species-specific differences. A previous study suggested that the time required for splicing and mRNA export from nuclei to cytoplasm accounts for the species-specific differences in the segmentation clock period (Hoyle and Ish-Horowicz, 2013). The iPSM method will be useful for testing these mechanisms directly in various species.

## MATERIALS AND METHODS

### Generation of mouse ES cells carrying *Hes7* and *Msgn1* reporters

The schematic structure of the *Hes7* and *Msgn1* reporters is indicated in Fig. 1A. This vector contained the *Hes7* promoter region (a 5377 bp fragment upstream of the first codon), cDNA sequences of Luc2 fused with human ubiquitin variant (G76V) and nuclear localization signal (Ub-NLS-Luc2) at the N-terminal end, and *Hes7* 3′-UTR. This vector also contained cDNA for histone H2B-mCherry fusion protein under the control of 1.2 kb *Msgn1* promoter (pMsgn) (Wittler et al., 2007). We used the Tol2 transposon system (a gift from the Kawakami lab, Mishima, Japan) (Kawakami, 2007; Yagita et al., 2010).

### ES cell culture and formation of iPSM tissues

All animals were handled in accordance with the Kyoto University's ‘Guide for the Care and Use of Laboratory Animals’. Mouse ES cells (E14TG2a, RBRC-AES0135 purchased from RIKEN Bio Resource Center; Hooper et al., 1987) were maintained without feeder cells in DMEM medium supplemented with 15% fetal bovine serum, 2 mM L-glutamine, 1 mM nonessential amino acids, 0.1 mM β-mercaptoethanol, 1 mM sodium pyruvate, penicillin, streptomycin, 1500 U/ml LIF, 3 µM CHIRON99021 and 1 µM PD0325901 with 5% CO_2_ at 37°C. To generate iPSM tissues, we used the method described previously (Chal et al., 2015) with some modifications. Briefly, ES cells were first cultured in DMEM/F12 medium supplemented with N2B27 reagent, 1% Knock-out Serum Replacement (KSR), 0.1% bovine serum albumin, 2 mM L-glutamine, 1 mM nonessential amino acids, 1 mM sodium pyruvate, penicillin, streptomycin and 10 ng/ml BMP4 (BMP medium) for 2 days. For induction of *Hes7* oscillations, it is very important to seed 1000 ES cells per well in low-cell-adhesion 96-well plates with U bottoms and culture these cells for 2 days. Cells were then cultured in gelatin-coated dishes with DMEM medium supplemented with 15% KSR, 2 mM L-glutamine, 1 mM nonessential amino acids, 1 mM sodium pyruvate, penicillin, streptomycin, 0.5% DMSO, 1 µM CHIRON99021 and 0.1 µM LDN193189 (CL medium).

### Time-lapse imaging and quantification of *Hes7* expression

For bioluminescence quantification, cells were plated in black 24-well plates and 0.5 mM luciferin was added to CL medium. Luciferase activity was recorded using a live-cell monitoring system equipped with a highly sensitive PMT (CL24B-LIC/B, Churitsu Electric). Photon-counting measurements were performed every 3 or 5 min with a 5 s exposure, and signals were obtained by counts per second (cps). The intensity was subjected to moving-average detrending (with a 36-frame temporal window). For imaging, cells were cultured in CL medium with 1 mM luciferin in gelatin-coated glass-based dishes with a 4-well micro-insert (FulTrac, Nippon Genetics). Alternatively, cells were plated onto fibronectin-coated glass-based dishes. Bioluminescence signals were captured using a cooled CCD camera (iKon-M 934, Andor) and analyzed using ImageJ software, as previously described (Imayoshi et al., 2013; Shimojo et al., 2016; Isomura et al., 2017). Stack images were applied to Spike-Noise Filter to remove signals from cosmic ray, and then Temporal Background Reduction was applied.

For making spatiotemporal profiles, sliced stack images were arranged from left to right in a temporal order. To obtain phase dynamics, images were subjected to moving-average detrending (with a 30-frame temporal window), then to Savitzky Golay filtering (with 41 frames temporal window) and finally to Hilbert transformation (Pikovsky et al., 2001). Quantification of periods of oscillations was carried out as previously described (Isomura et al., 2017). Briefly, peaks in the luminescence time series were identified by referring to the oscillator phases computed by the Hilbert transformation, and peak-to-peak intervals were counted to construct histograms of period distributions.

### *In situ* hybridization

*In situ* hybridization was performed, as described previously (Bessho et al., 2001). For dual-color fluorescent *in situ* hybridization, *Uncx4.1* probe was labeled with digoxigenin (DIG) using DIG RNA Labeling Mix (Roche), and *Hes7* probe was labeled with fluorescein using Fluorescein RNA Labeling Mix (Roche). After hybridization with these probes, samples were incubated with anti-DIG Fab fragments conjugated to horseradish peroxidase (HRP) and subjected to tyramide signal amplification (TSA-Cy3 for DIG-labeled probe, Perkin Elmer). Then, after inactivation of HRP by 3% H_2_O_2_, samples were incubated with anti-Fluorescein Fab fragments conjugated to HRP and subjected to tyramide signal amplification (TSA-Fluorescein for Fluorescein-labeled probe, Perkin Elmer).

### Quantitative RT-PCT (RT-qPCR)

cDNA was synthesized from total RNA using RNeasy Mini Kit (Qiagen). THUNDERBIRD SYBR qPCR Mix (TOYOBO) was used for real-time PCR with gene-specific primers (Table S1) and run on StepOnePlus (Applied Biosystems). β-Actin was used as an internal control. For reference, mouse E10.5 tissues were collected by dissecting the region from tail bud to somite-forming area (S0) as PSM.

### Bioluminescence imaging of the PSM explant culture

Explant culture of the PSM of *Hes7* reporter mice, pH7-UbLuc-In(−), was performed as described previously (Takashima et al., 2011). The PSM regions of reporter mice were put on glass-base dishes with 1 mM luciferin (Nacalai Tesque) in 1% BSA, 1 g/l glucose, 2 mM L-glutamine, 15 mM HEPES, penicillin, streptomycin, DMEM/F12 (Cell Culture Technologies) media and cultured in 80% O_2_ and 5% CO_2_ at 37°C. For image analysis, ImageJ software was used, as described previously (Niwa et al., 2011). Stack images were applied to a Spike-Noise Filter to remove signals from cosmic rays, and then Temporal Background Reduction was applied. Spatiotemporal profiles were obtained, as described above.

### Chemical treatment of iPSM colonies

A chemical library for epigenetics research (containing 80 compounds) was purchased from Sigma-Aldrich (S990043-EPI1). A single iPSM colony per well was cultured in gelatin-coated black 24-well plates, and each compound was added from day 4 onwards. *Hes7* promoter-driven luciferase activity was measured using a PMT.

## Supplementary Material

Supplementary information

Supplementary information
