# Supplementary material for: ES cell-derived presomitic mesoderm-like tissues for analysis of synchronized oscillations in the segmentation clock
Source: Development. 2018 Feb 15;145(4):dev156836. doi: 10.1242/dev.156836 (PMC5869006; doi:10.1242/dev.156836)
Supplement: Supplementary information [file develop-145-156836-s1.pdf]

Supplemental Information

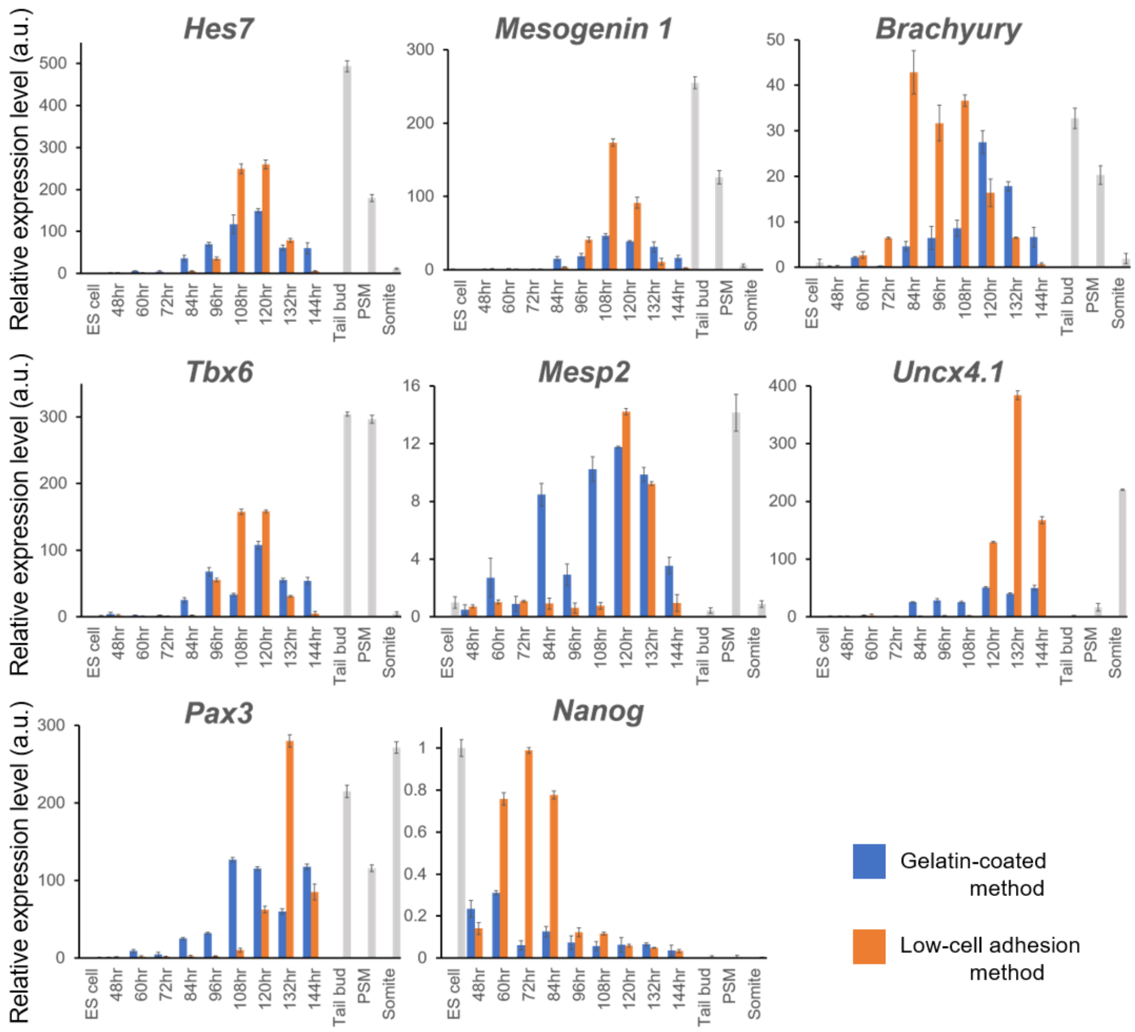

Figure S1, related to Figure 1. qPCR analyses of ES cell-derived PSM like cell cultures. Relative gene expression levels in cells cultured on gelatin-coated (blue, n = 6) and low-cell adhesion (orange, n = 6) plates were examined by qPCR at indicated time points. ES cells, and tail bud, PSM, and somite tissues of E10.5 mouse embryos were used as references. Each value represents the average of six independent experiments with a standard error.

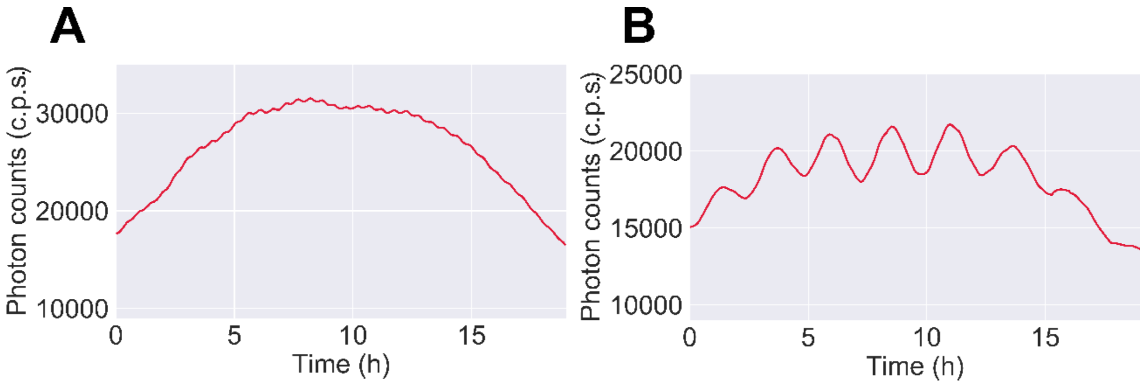

Figure S2, related to Figure 2. Time course of *Hes7* expression in ES cell-derived PSM-like cells. *Hes7* promoter-driven luciferase activity was measured by PMT from day 4 onward. Cells were cultured in BMP medium in gelatin-coated plates (A) or low-cell-adhesion plates (B) for two days and then transferred to CL medium in gelatin-coated plates. At least five independent samples per each group were examined, and all showed similar results. Only representative data are presented.

Development • Supplementary information

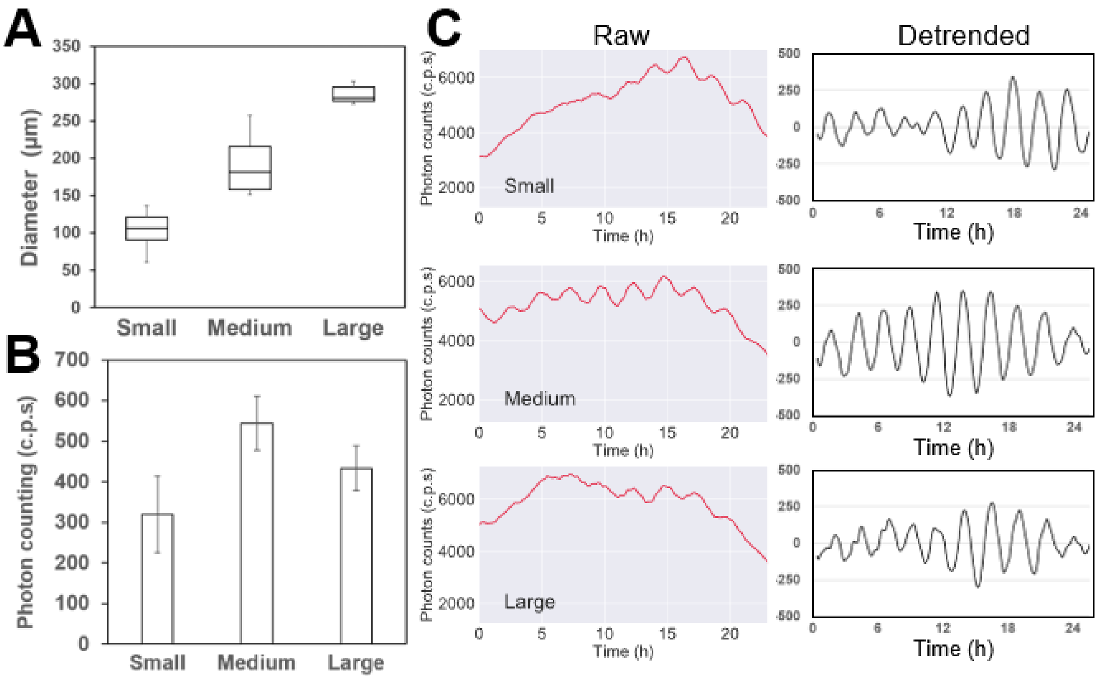

Figure S3, related to Figure 2. The effects of colony size on *Hes7* oscillations.

3,000 ES cells per well were plated in 96-well low-cell-adhesion plates with flat bottoms, and multiple colonies with various sizes were formed in each well after 2 days. These colonies were divided into three groups according to their size. (A) The average diameter of iPSM colonies of each group. (B) The average amplitudes of *Hes7* oscillations of each group. *Hes7* promoter-driven luciferase activities were measured by PMT from day 4 onward. The amplitudes of three highest pulses of detrended signals from each time series were measured and averaged. (C) Raw and detrended signals of *Hes7* expression of each group are shown. At least 15 independent samples per each group were examined, and all showed similar results. Only representative data are presented.

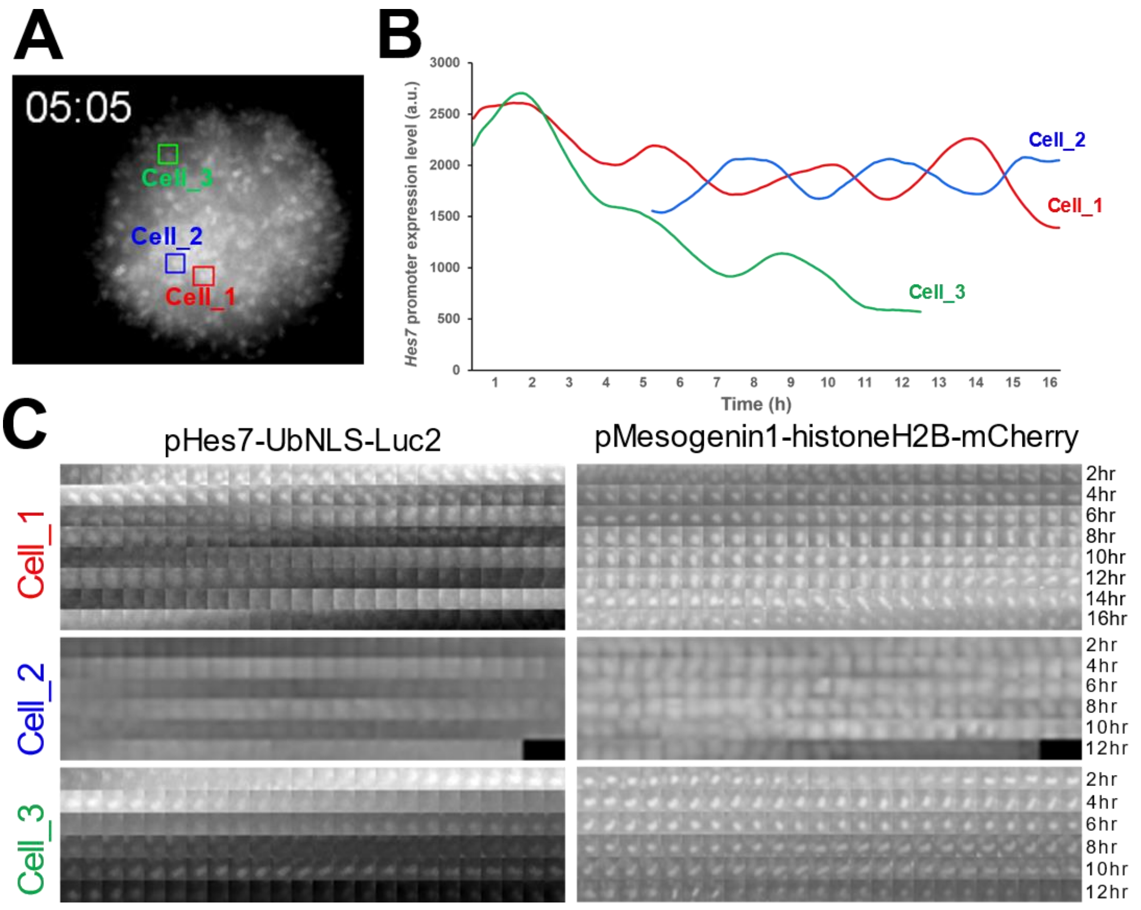

Figure S4, related to Figure 3. A mixed culture of an iPSM colony. An iPSM colony derived from mixtures of ES cells carrying the *Hes7* reporter and wild-type ES cells (1:150) were cultured in a fibronectin-coated dish, and live-imaging of *Hes7* expression was performed. 100-150 single cells, which were positive for *Hes7* reporter expression, were observed in this iPSM colony, but many of them actively moved, becoming out of focus. We were able to monitor 20 single cells over 8 h, and 18 of them showed oscillatory expression. (A) A snapshot of live-imaging of *Hes7* expression in an iPSM colony. Three representative cells, which were monitored over 8 h, are indicated. (B) Intensities of *Hes7* promoter-driven luciferase activities of three single cells. (C) Single-cell snapshots of *Hes7* promoter-driven luciferase activity and *Mesogenin* promoter-driven mCherry expression. *Hes7* promoter-driven luciferase activity was normalized by mCherry expression in (B).

Development • Supplementary information

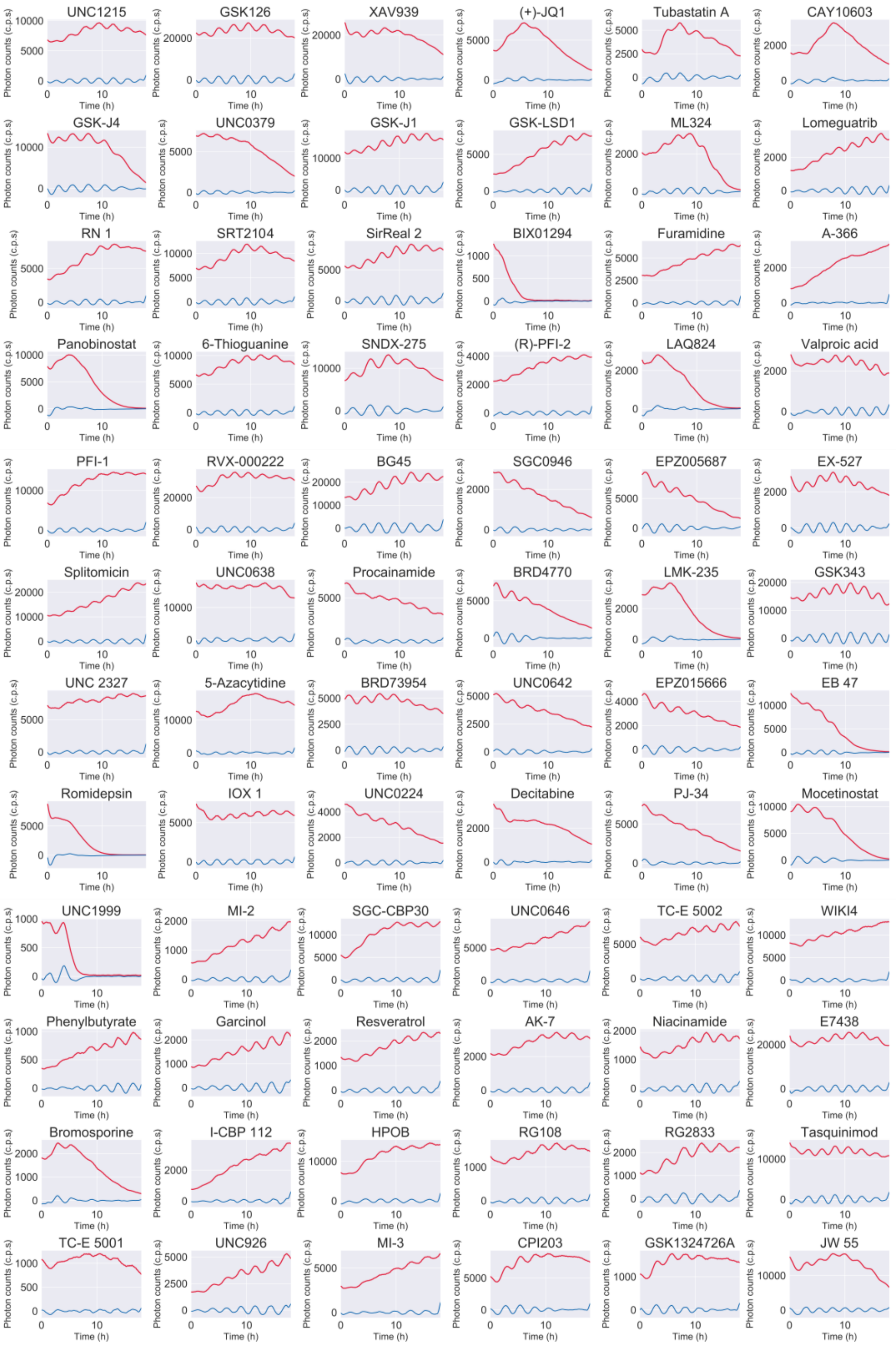

Figure S5, related to Figure 7. Chemical library screening with iPSM colonies. iPSM colonies were treated with 20µM of each chemical from day 4 onward. Raw (red) and detrended (blue) signals are shown. All chemicals were tested in duplicates and showed similar results. Only representative data are presented.

Primer name	Sequnces
Mesogenin1 Fwd	CTTCTGACACCGCTGGTCTG
Mesogenin1 Rev	GTGACTGCCGTAGCCATCG
Actin Fwd	GGCTGTATTCCCCTCCATCG
Actin Rev	CCAGTTGGTAACAATGCCATGT
Uncx4.1 Fwd	ACCCGCACCAACTTTACCG
Uncx4.1 Rev	TGAACTCGGGACTCGACCA
Tbx6 Fwd	ATGTACCATCCACGAGAGTTGT
Tbx6 Rev	CCAAATCAGGGTAGCGGTAAC
Mesp2 Fwd	GACTGGACACTGGACACAATCCACT
Mesp2 Rev	GGCCATAGCCAAGCAGACCTCAAA
Brachyury Fwd	CTGGGAGCTCAGTTCTTTCG
Brachyury Rev	GTCCACGAGGCTATGAGGAG
Hes7 Fwd	AGTATCTCCGCTTTCTCCAGCTT
Hes7 Rev	CATCAACCGCAGCCTAGAAGA
Nanog Fwd	AGGCTTTGGAGACAGTGAGGTGC
Nanog Rev	TACCCTCAAACTCCTGGTCCTTC

Table S1: Primer sequences used for RT-qPCR.

Movies

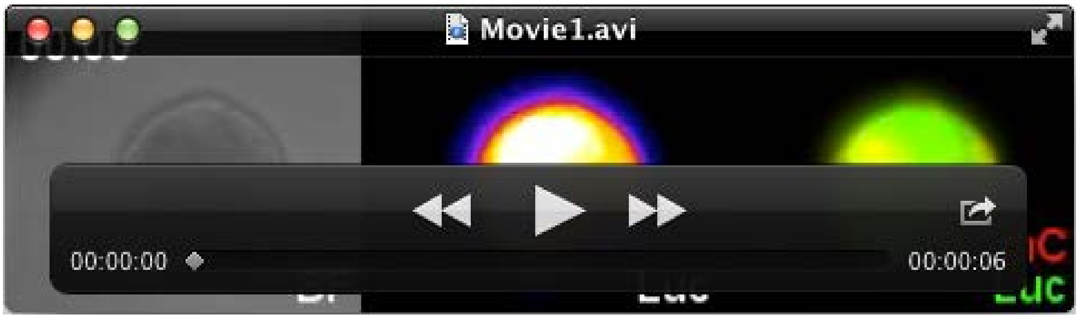

Movie 1. *Hes7* expression in an iPSM colony. Live-imaging of *Hes7* expression in a single iPSM colony cultured in a gelatin-coated plate. Bright field images (BF), the luciferase activity of Hes7 reporter (Luc), and *Mesogenin1* reporter expression (mC) were monitored.

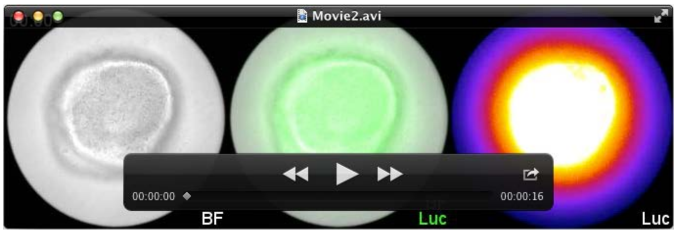

Movie 2. *Hes7* expression in an iPSM colony cultured in a fibronectin-coated plate. Bright field images (BF) and luciferase activity of Hes7 reporter (Luc) were monitored. Segmental boarder formation is indicated by arrowheads.

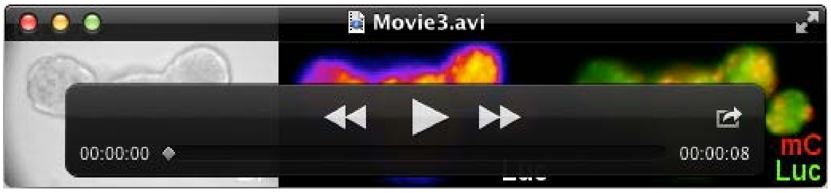

Movie 3. *Hes7* expression in a fused iPSM colony. Bright field images (BF), the luciferase activity of Hes7 reporter (Luc), and *Mesogenin1* reporter expression (mC) were monitored.

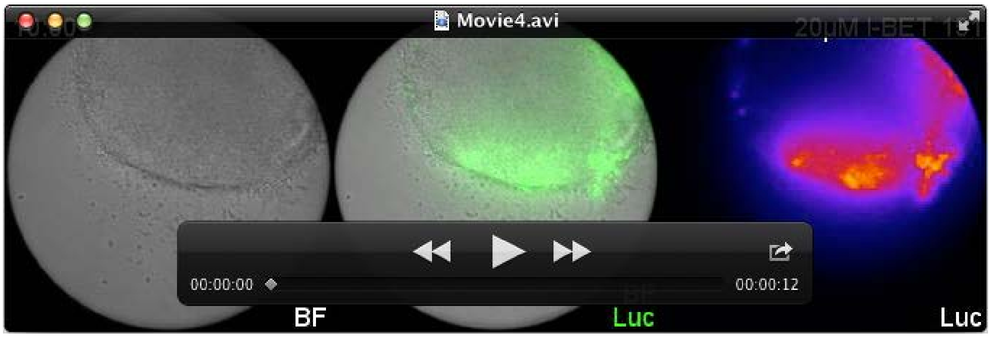

Movie 4. *Hes7* expression in an iPSM colony cultured in a fibronectin-coated plate in the presence of I-BET 151.

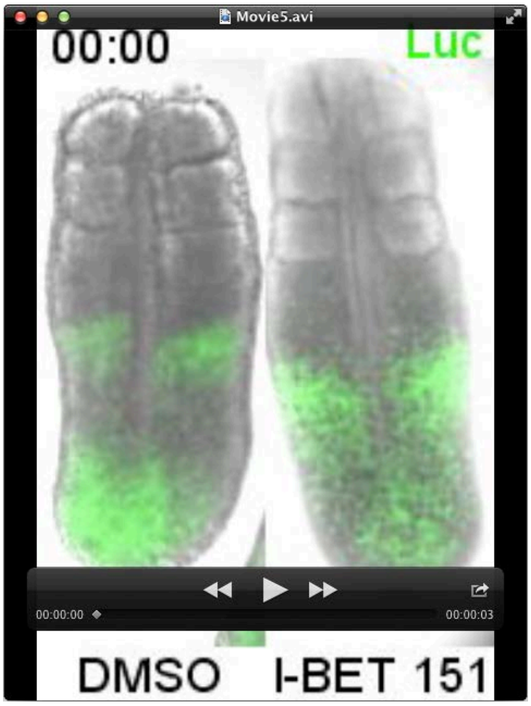

Movie 5. *Hes7* expression in explant cultures of the PSM of E10.5 mouse embryos carrying a *Hes7* reporter in the presence of either DMSO or I-BET 151. Newly formed somite boundaries are indicated by arrowheads.

Development • Supplementary information
